# Histone modification defects in developmental disorders and cancer

**DOI:** 10.18632/oncotarget.436

**Published:** 2012-01-25

**Authors:** Nicholas CP Cross

**Affiliations:** Salisbury District Hospital,Wessex Regional Genetics Laboratory

Covalent modifications of histone tails by methylation, acetylation and other changes play an important role in the regulation of gene expression, notably in the context of developmental decisions and cell fate. Two reports, including one in the December issue of *Oncotarget*, describe the use of whole exome sequencing to identify mutations in the histone modifying enzyme *EZH2* in Weaver syndrome, a rare developmental disorder characterised by generalised overgrowth, characteristic facial features and intellectual disability [1, 2]. The findings are remarkable because *EZH2* has strong credentials as either an oncogene or as a tumour suppressor in a variety of malignancies as a consequence of somatically acquired mutations.

EZH2 is the catalytic component of the polycomb repressive complex 2 (PRC2), which methylates histone H3 lysine 27, resulting in a mark (H3K27me3) that specifies a transcriptionally repressive chromatin environment. PRC2 consists of an additional two core components, SUZ12 and EED, which are required for complete function and stability of the complex. Elevated expression of *EZH2* has been reported in a number of epithelial and hematological malignancies and is associated with an adverse prognosis in prostate and breast cancer. Furthermore, overexpression has been causally linked to genomic deletion of microRNA-101 [3]. Consistent with an oncogenic role, monoallelic gain-of-function missense mutations at EZH2 tyrosine 641 have been found in B-cell lymphomas of germinal center origin that synergize with wild type EZH2 to result in increased levels of H3K27me3 [4]. In contrast, the *EZH2* mutations seen in myeloproliferative neoplasms (MPN) and myelodysplastic syndromes (MDS) are inactivating, dispersed throughout the gene, may be monoallelic or biallelic and are associated with an adverse prognosis [5]. The finding of both gain-of function and loss-of-function *EZH2* mutations is consistent with models suggesting that a critical balance of polycomb activity is essential for normal stem cell activity, with either loss or gain of polycomb function being potentially tumorigenic [6].

The mutations seen in Weaver syndrome patients are mostly missense substitutions or indels that preserve the reading frame, although three cases with truncating mutations involving the terminal exon were seen. Although not assessed functionally, several Weaver syndrome mutations are identical to some of the somatic changes described in MPN and MDS patients, strongly suggesting that they confer a loss-of-function. Despite this, only two of the 19 *EZH2*-mutated Weaver syndrome identified by Tatton-Brown and colleagues developed a malignant disorder [1]. Of these, one developed neuroblastoma and acute lymphoblastic leukemia at the age of 13 months and the second developed lymphoma at 12 years. It should be noted however that the eldest case in this series was only 27 years and that the natural history of Weaver syndrome remains under-investigated. It remains to be seen if there is a propensity to develop myeloid malignancies later in life. Clearly though, inherited *EZH2* mutations do not strongly predispose to early-onset myeloid malignancies, a fact that at first sight is somewhat surprising given the suggestion that acquisition of somatic *EZH2* mutations is an early event in the multistep pathogenesis of these disorders [5]. It will be important to determine if the mutations seen in Weaver syndrome are perhaps a relatively mild subset of mutations seen in MPN and MDS patients. In addition, inactivating mutations in *SUZ12* and *EED* are seen in hematological malignancies [7] and these genes are obvious candidates for *EZH2*-wild type cases of Weaver syndrome or related overgrowth disorders.

Clinically, Weaver syndrome is closely related to Sotos syndrome, which is frequently caused by mutations in *NSD1*. This gene also encodes a histone methyltransferase, in this case with activity against histone H3 lysine 36. *NSD1* is mutated in carcinoma of the upper aerodigestive tract (www.sanger.ac.uk/genetics/CGP/cosmic/) and also fuses to *NUP98* in acute myeloid leukemia. Looking more widely, whole exome screens in lymphoma, multiple myeloma, renal carcinoma and other malignancies have identified genes encoding diverse histone modifiers as targets of somatic mutation. Strikingly, several of these (e.g. *MLL2*, *EP300*, *CREBBP, ASXL1*) are also mutated in human developmental disorders thus pointing towards a remarkable and unexpected convergence between somatic and germline genetics.
